# Solitary Fibrous Tumour Presenting as Delayed Neck Swelling Following Carotid Endarterectomy

**DOI:** 10.7759/cureus.94284

**Published:** 2025-10-10

**Authors:** Yousif Jihad, Timothy J Belza, Peter Lee Chong

**Affiliations:** 1 Surgery, United Lincolnshire Teaching Hospitals NHS Trust, Boston, GBR; 2 Vascular Surgery, United Lincolnshire Teaching Hospitals NHS Trust, Boston, GBR

**Keywords:** anterior neck mass, carotid artery surgery, general and vascular surgery, oncology imaging, post carotid endarterectomy, sarcoma soft tissue, sft, solitary fibrous tumor (sft), solitary fibrous tumour (sft)

## Abstract

Solitary fibrous tumours are rare mesenchymal neoplasms that can arise in a variety of anatomical sites, including the head and neck. We present an unusual case of an elderly man who developed a slowly enlarging, painless swelling in the neck one year after undergoing carotid endarterectomy. Initial suspicion of a delayed post-operative complication was revised after imaging and biopsy confirmed a solitary fibrous tumour extending into the mediastinum. Due to the lesion’s size, anatomical complexity, and the patient’s comorbidities, surgical resection was not feasible, and he was referred for palliative oncological management. This case underscores the importance of considering rare neoplastic causes in delayed post-surgical swellings and highlights the role of a thorough histopathological evaluation in establishing a definitive diagnosis.

## Introduction

Solitary fibrous tumours (SFTs) are rare mesenchymal neoplasms characterised by spindle cell morphology and a collagen-rich stroma [1,2]. They were originally described in the pleura but are now recognised in a wide range of anatomical sites, including the abdomen, extremities, and head and neck region [3]. These tumours are typically slow-growing, but a subset demonstrates aggressive behaviour with recurrence and metastasis [4]. Diagnosis is difficult, as imaging findings often overlap with more common pathologies, such as haematomas or seromas, and confirmation relies on histopathological analysis with immunohistochemical markers including CD34 and STAT6 [1,5]. Head and neck SFTs remain particularly uncommon, and their presentation can mimic post-surgical complications, which may delay recognition [2,3]. We present an unusual case of a solitary fibrous tumour arising one year after carotid endarterectomy, which, in this instance, demonstrated rapid growth and malignant potential, highlighting the diagnostic challenges and the need for thorough evaluation in patients presenting with delayed neck swelling.

## Case presentation

An 85-year-old male presented to the emergency department with a gradually enlarging swelling on the left side of his neck, most prominent beneath the lower half of a healed surgical scar along the anterior margin of the sternocleidomastoid muscle. Twelve months earlier, he had undergone a left carotid endarterectomy due to symptomatic left internal carotid artery stenosis. His early postoperative course was uneventful. At a routine follow-up two weeks after surgery, the wound was healing well, with no evidence of swelling. A routine postoperative carotid Doppler scan confirmed a widely patent left internal carotid artery, no significant disease on the right side, forward flow in both vertebral arteries, and a small non-vascular collection above the left carotid artery.

The patient first noticed the swelling six months post-surgery, which progressively increased in size over time. He reported no associated pain, dysphagia, or respiratory symptoms. Four days before he visited the hospital, he began experiencing new-onset lethargy and intermittent dizziness, prompting hospital attendance.

Clinical examination revealed a non-tender, soft-to-firm, non-pulsatile mass, approximately 4 cm in diameter, located beneath the surgical scar, with normal overlying skin. There was no palpable thrill or audible bruit, and the systemic examination was otherwise unremarkable.

**Figure 1:**
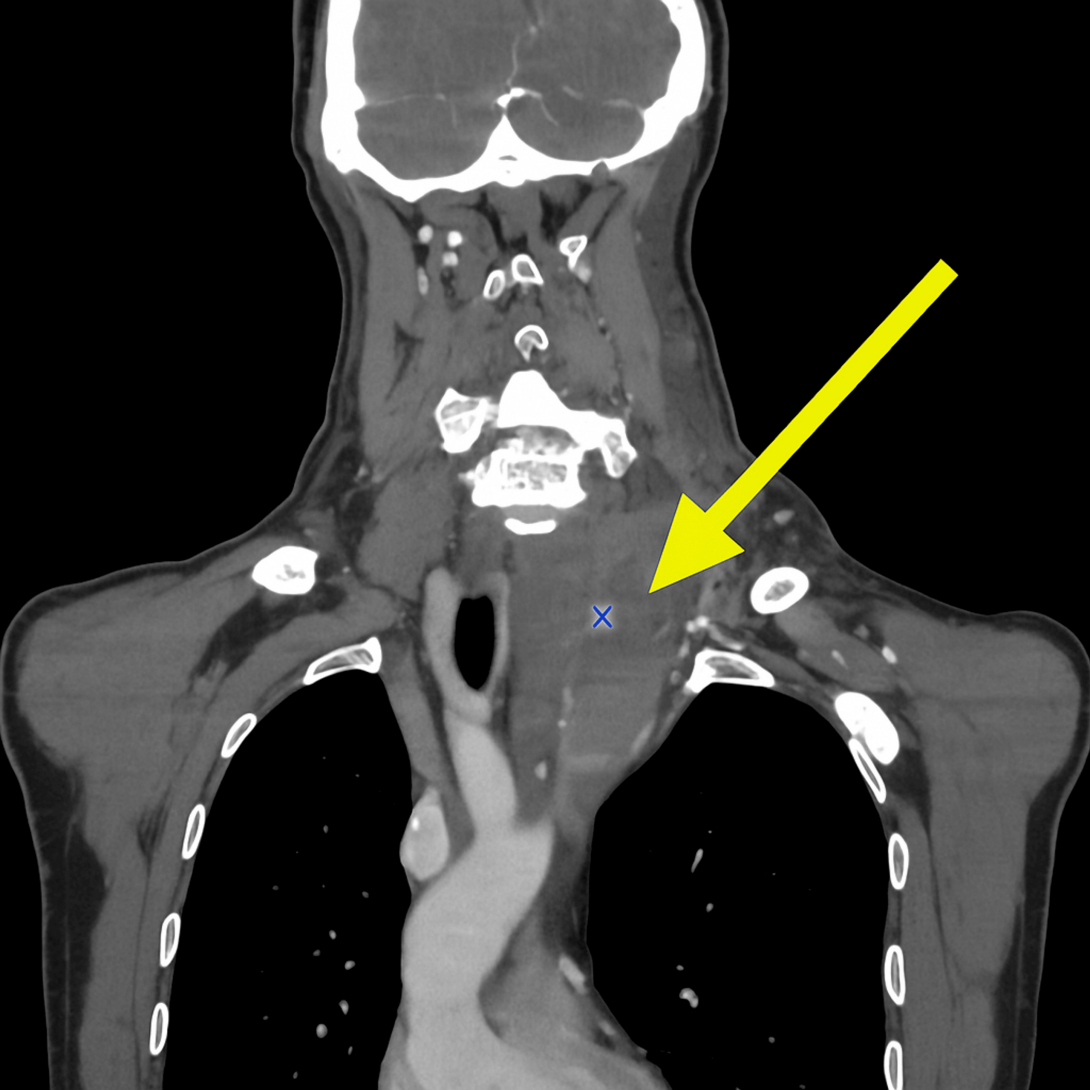
Contrast-enhanced CT scan of the neck (coronal view) demonstrating a well-defined, soft-tissue mass (yellow arrow) located in the lower cervical region. Scanned locally within Pilgrim Hospital, United Lincolnshire Teaching Hospitals NHS Trust

Laboratory investigations showed mild anaemia (haemoglobin 11.9 g/dL) and elevated inflammatory markers (WBC 13 x 10^9/L, neutrophils 11.15 x 10^9/L, and C-reactive protein (CRP) 64 mg/L). The clotting profile was normal. An initial CT angiogram (Figure 1) reported a heterogeneous collection in the left neck measuring 9.7 x 7.2 x 5.8 cm, extending from the level of the C4 vertebra to the aortic arch. Differential diagnoses included late-onset postoperative hematoma, seroma, abscess or post-operative infection, recurrent or residual soft tissue collection, primary soft tissue tumour (e.g., lipoma, sarcoma), or solitary fibrous tumour (whether de novo or surgery-associated). There was no evidence of a pseudoaneurysm.

**Figure 2:**
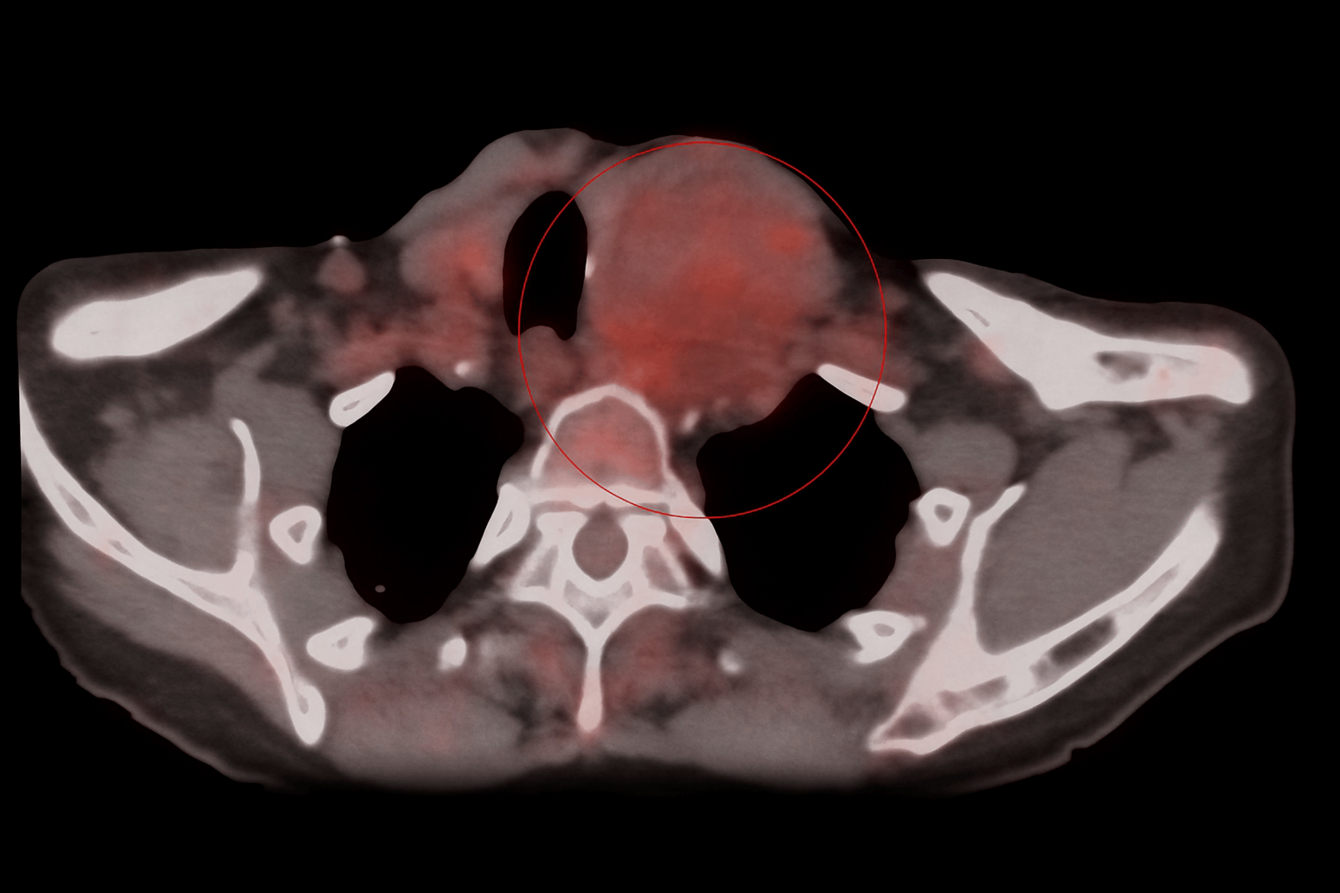
Axial PET-CT image of the neck demonstrating a soft tissue mass in the left lower cervical region with increased heterogeneous tracer uptake Scanned locally within Pilgrim Hospital, United Lincolnshire Teaching Hospitals NHS Trust PET-CT: positron emission tomography-computed tomography

A further contrast-enhanced CT scan of the chest, abdomen, and pelvis showed no other abnormalities. Duplex ultrasound confirmed good blood flow in the carotid arteries bilaterally with no re-stenosis. Magnetic resonance angiography (MRA) demonstrated a large, heterogeneously enhancing mass extending from the lower neck into the superior mediastinum, without splaying of the carotid bifurcation. Positron emission tomography (PET)-computed tomography (CT) revealed increased tracer uptake within the lesion, consistent with malignancy, but no nodal or distal metastasis (Figure 2).

Ultrasound-guided core needle biopsy using an 18-gauge BioPince instrument was performed. Initial histology suggested a malignant spindle cell tumour of possible neural origin. Subsequent specialist review confirmed a diagnosis of solitary fibrous tumour. Microscopy showed a hypercellular spindle cell neoplasm with haphazardly arranged ovoid nuclei and fibrous stroma, with areas of ectatic vessels and necrosis.

**Figure 3:**
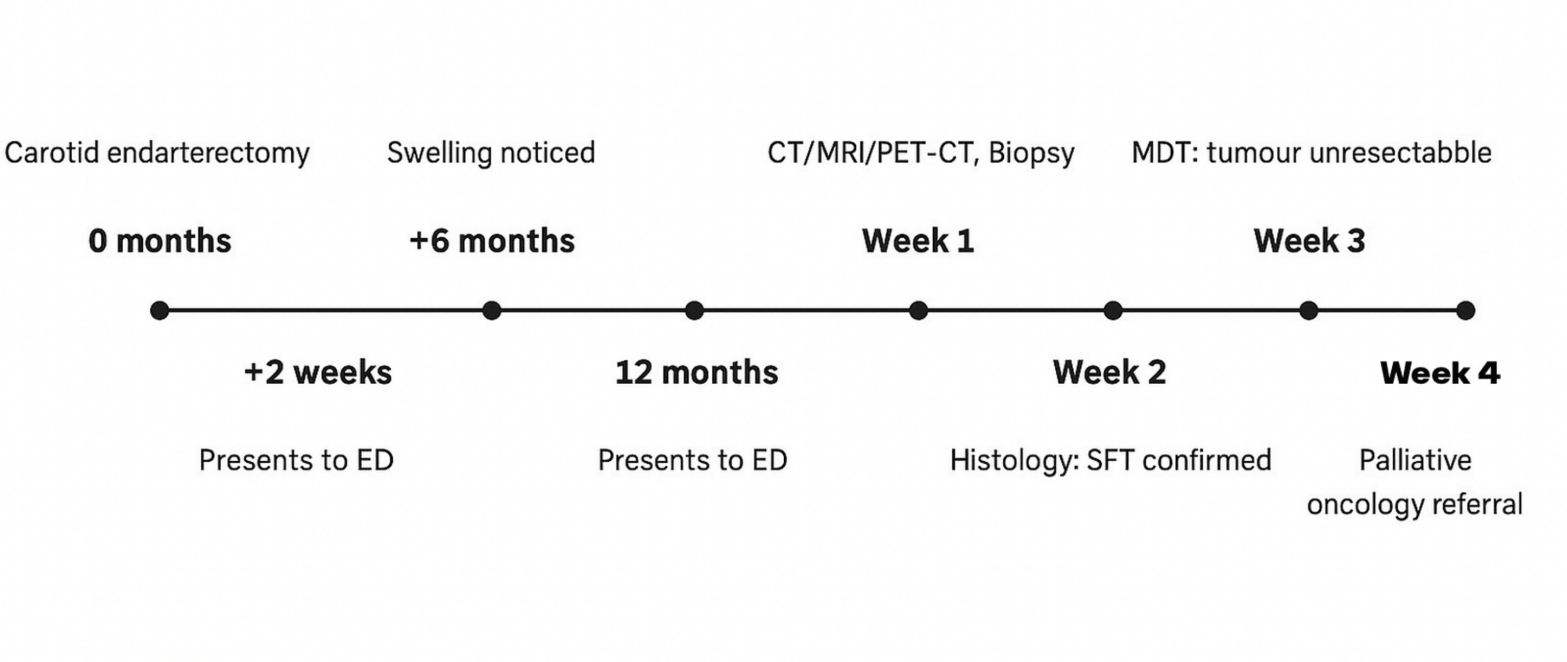
Timeline of key clinical events from surgery to diagnosis and palliative referral

Immunohistochemistry was positive for CD34, STAT6, TLE1 and CD56, supporting the diagnosis. The tumour was classified as intermediate risk. Following a multidisciplinary team discussion (MDT) at the Regional Sarcoma MDT, the lesion was deemed nonresectable due to its size, anatomical location, and proximity to major vascular structures. Given the patient’s age and surgical risk, curative resection was not advised. The patient was referred for palliative oncological treatment (Figure 3).

## Discussion

Solitary fibrous tumours (SFTs) are rare mesenchymal neoplasms [1,2], most commonly arising from the pleura, though they may develop in virtually any anatomical location, including the head and neck. While generally benign, a subset exhibits malignant behaviour. SFTs account for fewer than 2% of all soft tissue tumours and are typically diagnosed in middle-aged adults of either sex. Clinical presentation varies depending on tumour location, with patients presenting with a palpable mass or incidental radiological findings.

The pathogenesis of SFTs remains incompletely understood, although a recurrent NAB2-STAT6 gene fusion has been identified as a characteristic molecular alteration. This fusion gene results from a chromosomal inversion and plays a crucial role in tumorigenesis. No definitive environmental or genetic risk factors have been established [3].

Diagnosis is challenging due to non-specific imaging features. While CT, MRI, and PET scans aid in localisation and staging, histological analysis remains the gold standard. Microscopically, SFTs are composed of randomly arranged spindle or ovoid cells within a collagen-rich stroma and may show characteristic ‘staghorn’ vasculature. Immunohistochemistry typically demonstrates positivity for CD34, STAT6, Bcl-2, and CD99, supporting the diagnosis [4].

Complete surgical resection with negative margins is the preferred curative treatment. The role of adjuvant therapy remains uncertain and is generally reserved for high-risk, recurrent, or incompletely resected tumours. Radiotherapy may be considered in unresectable cases, while chemotherapy and targeted molecular therapies (e.g. NAB3-STAT6 inhibitors) are investigational and used in select cases with metastatic or progressive disease [5].

Prognosis is variable and depends on histological features and risk stratification models. In the present case, the tumour’s size, anatomical complexity, and patient comorbidities precluded surgical resection, necessitating a palliative approach with radiotherapy to reduce the tumour burden and size and regular follow-ups at the oncology outpatient clinic.

## Conclusions

This case highlights a rare presentation of a solitary fibrous tumour arising at the site of previous carotid surgery. The speed at which the lesion presented following surgical endarterectomy of the left carotid artery suggests an aggressive and rapidly growing tumour. While the temporal and anatomical relationship raises the question of a potential association, it remains unclear whether this was coincidental or triggered by surgical intervention. Further research is required to better elucidate the aetiology, natural history, and optimal management strategies for these uncommon tumours.
